# Cost-effectiveness of 2 + 1 dosing of 13-valent and 10-valent pneumococcal conjugate vaccines in Canada

**DOI:** 10.1186/1471-2334-12-101

**Published:** 2012-04-24

**Authors:** Stephanie R Earnshaw, Cheryl L McDade, Giovanni Zanotti, Raymond A Farkouh, David Strutton

**Affiliations:** 1 RTI Health Solutions, 200 Park Offices Drive, Research Triangle Park, NC, 27709, USA; 2 Pfizer Canada Inc., 17300 Trans Canada, Kirkland, Quebec, H9J 2M5, Canada; 3 Pfizer Inc., 500 Arcola Road, Collegeville, PA, 19426, USA

**Keywords:** Vaccine, Cost-effectiveness, Pneumococcal conjugate vaccine, Pneumococcal disease

## Abstract

**Background:**

Thirteen-valent pneumococcal conjugate vaccine (PCV13) and 10-valent pneumococcal conjugate vaccine (PCV10) are two recently approved vaccines for the active immunization against *Streptococcus pneumoniae* causing invasive pneumococcal disease in infants and children. PCV13 offers broader protection against *Streptococcus pneumoniae*; however, PCV10 offers potential protection against non-typeable *Haemophilus influenza* (NTHi). We examined public health and economic impacts of a PCV10 and PCV13 pediatric national immunization programs (NIPs) in Canada.

**Methods:**

A decision-analytic model was developed to examine the costs and outcomes associated with PCV10 and PCV13 pediatric NIPs. The model followed individuals over the remainder of their lifetime. Recent disease incidence, serotype coverage, population data, percent vaccinated, costs, and utilities were obtained from the published literature. Direct and indirect effects were derived from 7-valent pneumococcal vaccine. Additional direct effect of 4% was attributed to PCV10 for moderate to severe acute otitis media to account for potential NTHi benefit. Annual number of disease cases and costs (2010 Canadian dollars) were presented.

**Results:**

In Canada, PCV13 was estimated to prevent more cases of disease (49,340 when considering both direct and indirect effects and 7,466 when considering direct effects only) than PCV10. This translated to population gains of 258 to 13,828 more quality-adjusted life-years when vaccinating with PCV13 versus PCV10. Annual direct medical costs (including the cost of vaccination) were estimated to be reduced by $5.7 million to $132.8 million when vaccinating with PCV13. Thus, PCV13 dominated PCV10, and sensitivity analyses showed PCV13 to always be dominant or cost-effective versus PCV10.

**Conclusions:**

Considering the epidemiology of pneumococcal disease in Canada, PCV13 is shown to be a cost-saving immunization program because it provides substantial public health and economic benefits relative to PCV10.

## Background

A 7-valent pneumococcal polysaccharide-protein conjugate vaccine, Prevenar (PCV7), licensed for use in Canada in 2001 in children, has led to a dramatic decrease in *Streptococcus pneumoniae*-related diseases such as acute otitis media (AOM), pneumonia (PNE), and invasive pneumococcal disease (IPD) in Canada [1-8]. Two pneumococcal conjugate vaccines have recently been approved for use in infants in Canada. The 10-valent pneumococcal conjugate vaccine (PCV10) [9] offers seroprotection against the original seven pneumococcal serotypes plus three additional serotypes 1, 5, and 7F. The 10 serotypes are conjugated to three different proteins, one of which is protein D, which may provide protection against non-typeable *Haemophilus influenzae* (NTHi) [10]. The 13-valent pneumococcal conjugate vaccine Prevenar 13 (PCV13) provides the same seroprotection as PCV7 [11] and contains the 10 serotypes in PCV10 plus three additional pneumococcal serotypes: 3, 6A, and 19A [11]. PCV10 was originally approved with a 3 + 1 dosing schedule but was recently approved for a 2 + 1 dosing schedule in Europe [12]; it previously was used in Quebec with a 2 + 1 schedule [9]. As of January 2011, after Ontario and Quebec switched from PCV10 to PCV13, all provinces in Canada currently are using PCV13 in their immunization programs. Understanding the value for money or cost-effectiveness of vaccine policies with these newer vaccines is important.

Recently published analyses have examined the cost-effectiveness of PCV10 compared with PCV7 in Canada [13]. Analyses comparing the use of PCV13 with PCV10 in the provinces of Alberta [14] and Quebec [15,16] also have been performed. Although Chuck et al. and Roussy et al. examined the value of PCV10 with a 2 + 1 dosing schedule, the Canadian-specific analysis by Talbird et al. examined the use of PCV10 with a 3 + 1 dosing schedule. In the current analysis, we expanded on previously published analyses by comparing costs and outcomes associated with the use of PCV13 compared with PCV10 using a two-dose primary series followed by a booster dose at 12 to 15 months across all provinces and territories in Canada.

## Methods

### Model design

**Figure 1:**
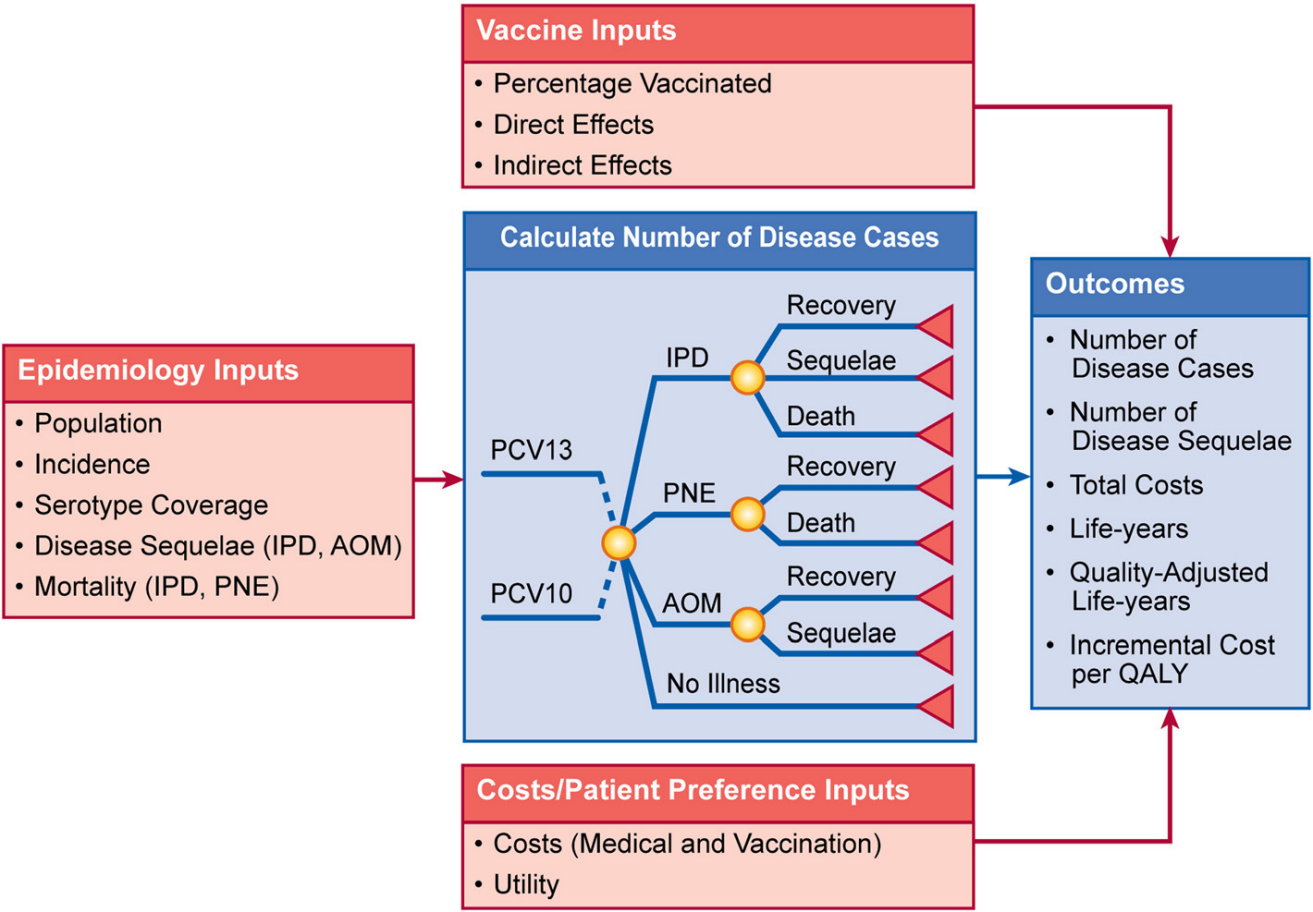
**Model structure.** AOM = acute otitis media; IPD = invasive pneumococcal disease; PCV10 = 10-valent pneumococcal conjugate vaccine; PCV13 = 13-valent pneumococcal conjugate vaccine; PNE = pneumonia; QALY = quality-adjusted life-year.

A decision-analytic model (Figure 1) was constructed to examine the costs and outcomes of vaccine policies that included PCV13 and PCV10 for vaccinating children against *Streptococcus pneumoniae*, which causes IPD (bacteremia and meningitis), PNE (hospitalized and ambulatory), and AOM (mild and moderate/severe). The model was programmed in Microsoft Excel version 2003.

Individuals entered the model either vaccinated or not vaccinated, depending upon their ages and vaccination uptake. Individuals were followed over the remainder of their lifetime, in which they were at risk for contracting IPD, PNE, or AOM based on their vaccination status. If individuals contracted IPD, they had a risk of developing sequelae such as hearing loss and/or neurological impairment or dying from IPD. Individuals contracting PNE had a risk of dying if they were hospitalized. Children who contracted moderate/severe AOM were at risk for requiring myringotomy.

Cases of disease were derived from population, epidemiology, and clinical data from the published literature. Specific data included Canadian populations, incidence of disease, serotype coverage, and direct and indirect effects associated with each vaccine. Based on the estimated disease cases, mortality and disease sequelae were estimated. Costs and utilities from the published literature were applied to derive the overall costs and health benefits for individuals on each vaccine. Costs and outcomes were presented from a Canadian health system perspective. Costs were reported in 2010 Canadian dollars.

### Population

The model examined the impact of implementing pediatric vaccine policies, including PCV13 and PCV10, among individuals of all ages in Canada. Specifically, 34,108,000 individuals within Canada were considered [17]; of these, 1.4% or 477,512 children < 2 years of age were considered for the vaccinated cohort. In the base case, we assumed 91% of these children would be vaccinated [2]. Vaccination uptake was examined in sensitivity analysis, where an uptake of 87% was examined [18]. The remaining individuals comprised the non-vaccinated cohorts, which included individuals aged 2 to < 5, 5 to < 18, 18 to < 65, and 65+ years.

### Inputs

Model inputs are categorized as epidemiological, vaccine-related, and economic inputs. Each is discussed separately.

### Epidemiology

#### Disease incidence

As a result of the introduction of PCV7, a dramatic decrease in the incidence of PCV7-typed disease has occurred over time [2,18,23,24]. Thus, time since the introduction of PCV7 is a significant influencer of incidence in a population. Because of the limited availability of data and the need for use of the most current data, the incidence of IPD for Canada was estimated from the “Programme de surveillance du pneumocoque du Quebec” [18] From this report, the number of IPD cases by age were comprised of 88% bacteremia (isolated from blood) and 3.1% meningitis (isolated from cerebrospinal fluid) [18], consistent with previously published cost-effectiveness research [15]. Incidence of IPD from Morrow et al. [19] was examined in sensitivity analysis as the Morrow et al. [19] data are all-Canada specific.

Incidence of PNE was estimated from Morrow et al., Petit et al., and Nelson et al. [19-21]. Specifically, Nelson et al. [21] examined the impact of introducing a 7-valent pneumococcal conjugate vaccine for children and adults in the United States. Within this study, the authors calculated incidence rate ratios comparing a period of time after (2003–2004) with a period before (1998–2000) the introduction of the national immunization program. Using these incidence rate ratios for outpatient and hospitalized PNE, the incidence of pneumococcal PNE as reported by Morrow et al. [19] for Canada was adjusted. All-cause PNE then was estimated by multiplying by the ratio of pneumococcal PNE to all-cause PNE as reported by Petit et al. [20].

Incidence of AOM was estimated from the number of AOM claims reported in Quebec in 2007 [22]. In this study, the authors reported 44 claims per 100 person-years, of which 13.5% were seen by an ear, nose, and throat specialist [22]. The model assumed ear, nose, and throat specialist claims as moderate/severe AOM and all other claims as mild AOM. Incidence was further distributed by age based on the monthly frequency of physician claims by age [22]. Incidence of AOM from Morrow et al. [19] was examined in sensitivity analysis. Disease incidence by age is presented in Table 1.

**Table 1:** Input parameters

**Parameters**	**Age (Years)**					**Source(s)/Assumption**
	**0–2**	**2–4**	**5–17**	**18–64**	**65+**	
Incidence (per 100,000)						
Bacteremia	14.77	10.62	6.74	43.67	87.07	[18]
Meningitis	0.52	0.37	0.24	1.54	3.07	
Inpatient PNE	1,135.13	380.99	170.84	102.76	1,343.40	[19-21]
Outpatient PNE	874.77	460.69	514.63	55.35	63.31	
Mild AOM	13,762.21	13,811.12	10,486.67	0.00	0.00	[22]
Moderate/severe AOM	2,147.86	2,155.49	1,636.65	0.00	0.00	
Serotype coverage						
PCV10	0.18	0.23	0.26	0.28	0.22	[23]
PCV13	0.61	0.68	0.42	0.55	0.55	
Mortality						
Bacteremia	0.01	0.003	0.003	0.07	0.11	[26-28]
Meningitis	0.02	0.003	0.003	0.07	0.11	
Inpatient PNE	0.01	0.003	0.003	0.07	0.11	
Direct medical costs						
Bacteremia	$10,578.71	$3,379.85	$9,768.95	$13,559.64	$11,913.26	[19,51]
Meningitis	$48,382.04	$24,615.09	$61,114.72	$22,409.11	$11,782.22	
Inpatient PNE	$3,450.13	$2,149.73	$10,305.00	$9,606.02	$8,370.61	
Outpatient PNE	$138.18	$69.09	$184.24	$126.66	$103.63	
Mild AOM	$95.30	$47.65	$71.47	$0.00	$0.00	
Moderate/severe AOM	$95.30	$47.65	$71.47	$0.00	$0.00	
Utilities	0.97	0.97	0.97	0.88	0.82	[54]

*AOM* acute otitis media; *PCV10* 10-valent pneumococcal conjugate vaccine; *PCV13* 13-valent pneumococcal conjugate vaccine; *PNE* pneumonia.

#### Serotype coverage

Serotype coverage for IPD for PCV10 and PCV13 were obtained from the National Centre for Streptococcus, from which isolates from all Canadian territories (excluding Quebec) were collected over the years [23]. Vaccine-specific serotype coverage was obtained by age for 2009, the most current available year. These data are presented in Table 1. We did not consider cross-protection to 6A or 19A for PCV10 because there was limited evidence of vaccine effectiveness against either potentially cross-reactive serotype.

Sensitivity analyses were performed using serotype coverage obtained from the Quebec surveillance program [18] from 2009 and the Calgary Area *Streptococcus pneumoniae* Epidemiology Research group from 2007 [2]. Sensitivity analyses also were performed with serotype coverage estimated by Bettinger et al., in which isolates from 12 centers of the Canadian Immunization Monitoring Program ACTive were examined [24,25]. These data also were used within the analyses performed by Talbird et al. [25]. All sensitivity analysis on serotype coverage data are presented in Additional file 1: Table A1. We also examined the impact of increasing serotype coverage of PCV10 relative to PCV13 to account for uncertainty in the serotype coverage data.

PNE and AOM were considered from an all-cause perspective because etiology is typically not determined for cases of mucosal disease. Thus, serotype coverage data were implicitly considered within vaccine effectiveness.

#### Mortality

General mortality in individuals was assumed to be similar to that observed in the general Canadian population [26]. IPD-specific and inpatient PNE mortality was obtained from Scheifele et al. [27] and Jette et al. [28] and is presented in Table 1. Deaths due to outpatient PNE and AOM were assumed to not occur.

### Direct effects of vaccines

Direct effects are the direct reductions in disease cases that occur in individuals due to the individual being vaccinated. These effects are specific to the covered serotypes for IPD and are all-cause for PNE and AOM. As of the writing of this article, efficacy and effectiveness data for PCV13 and PCV10 were not available. However, interim analyses of surveillance data and efficacy trials have been presented [29,30], and full data and details are expected to be available in the future. In the absence of such data, the effects for these vaccines were derived from the direct effects observed by individuals vaccinated with PCV7, a similar approach utilized by previous analyses [13,14,25,31,32]. Specific direct effects for each vaccine in the different diseases are discussed next.

Direct effects for PCV7 for preventing IPD among covered serotypes was reported to be 0.94 by Black et al. [33] We assumed that direct effects on covered serotypes experienced by individuals vaccinated with PCV10 or PCV13 would be similar to those experienced by individuals vaccinated with PCV7. Deceuninck et al. [34] performed a study of children receiving PCV7 in a 2 + 1 dosing schedule in Quebec; the authors reported an effectiveness of 0.92 on IPD cases. We examined the effects of this reduced effectiveness in sensitivity analysis.

When using the Percentages of Children Achieving the Serotype-Specific Antibody Threshold of 0.2 μg/mL, as Measured 1 Month After Completion of the Primary Series as a criteria for comparison (i.e., criteria defined by World Health Organization) the immunogenicity data for PCV10 was non-inferior to PCV7 for serotypes 4, 9 V, 14, 18 C, and 19 F [35], However, other response measures of PCV10 against common serotypes with PCV7 such as geometric mean concentrations of serotype-specific antibodies, as measured 1 month after completion of the primary series indicated a potentially lower response to PCV10 compared with PCV7. As a result, we examined the potential effect of lower immunogenic response in sensitivity analysis, in which direct effects for IPD, inpatient PNE, outpatient PNE, and all AOM were reduced to 0.90, 0.85, 0.80, and 0.75 of the serotype extrapolated direct effects, respectively. Larger reductions in direct effects for PNE and AOM than for IPD were assumed because attaining seroprotection against mucosal disease was more difficult than against IPD [36]. Lower immunogenic response for PCV13 was not assumed because PCV13 shares the same carrier protein as PCV7 and has a comparable immunogenic response [37-39].

Direct effects in preventing PNE and AOM were obtained for all-cause PNE and all-cause AOM. Direct effects of PCV7 in preventing inpatient PNE was estimated at 0.26 [40]. Outpatient PNE was estimated at 0.06 [41]. Although PCV10 and PCV13 covered more serotypes than PCV7, the serotype coverage of PCV7 when it was introduced was greater than the potential current serotype coverage of both PCV13 and PCV10. Therefore, the direct effects for PCV10 and PCV13 were assumed to be similar to PCV7 but then adjusted downward based on PCV7’s coverage prior to PCV7 introduction relative to PCV10’s or PCV13’s current serotype coverage. Thus, PCV13 was assumed to have 0.19 and 0.04 direct effects for inpatient and outpatient PNE, respectively. PCV10 was assumed to have 0.05 and 0.01 direct effects for inpatient and outpatient PNE respectively.

Direct effects of PCV7 in preventing mild AOM and moderate/severe AOM was estimated at 0.07 [40] and 0.15 [42], respectively. Similar to PNE, direct effects for PCV10 and PCV13 were derived by adjusting the PCV7 direct effects by serotype coverage pre-introduction of PCV7 relative to PCV10’s or PCV13’s current serotype coverage. Thus, PCV13 was assumed to have 0.05 and 0.11 direct effects for mild and moderate/severe AOM, respectively. PCV10 was assumed to have 0.01 and 0.03 direct effects for mild and moderate/severe AOM, respectively. The direct effects for PCV10 in moderate/severe AOM were further increased to account for potential coverage of NTHi. Specifically, in the POET trial [10], per-protocol vaccine efficacy against NTHi AOM was reported as 31.1%. The etiology of NTHi in the control group was 63 NTHi cases out of 499 AOM cases. Therefore, the reduction in AOM due to NTHi was estimated as an absolute increase in effectiveness of 0.04 (31.1% × 63/499). It was assumed that AOM etiology in POET would be similar to that observed in the moderate/severe cases in Canada.

### Indirect effects

Indirect effects, or herd protection, occur when an unvaccinated individual does not contract pneumococcal disease because other individuals who have been vaccinated are less likely to carry vaccine-type pneumococci [43]. Robust indirect effects have been demonstrated for PCV7 national immunization programs where the seven serotypes in PCV7 have been reduced dramatically [44-47]. Indirect effects for PCV7 are presented in Table 2. Indirect effects for PCV13 were assumed to occur because PCV13 shares the same carrier protein as PCV7, has demonstrated a similar immune response to PCV7 [37-39], and has shown a statistically significant reduction in nasopharyngeal carriage [48]. In addition, a recent study by the Centers for Disease Control (CDC) has demonstrated that PCV13-type IPD has declined in adults (i.e., nonvaccinated individuals) in the US [49]. As a result, similar to estimating direct effects for PNE and AOM, indirect effects for PCV13 were derived by adjusting the PCV7 indirect effects by serotype coverage pre-introduction of PCV7 relative to PCV13 current serotype coverage. Indirect effects in the presence of PCV10 were assumed to not occur in the base-case analysis because the 11-valent investigational vaccine that preceded PCV10 and PCV10 did not show a consistent statistically significant effect on pneumococcal nasopharyngeal carriage [10,50,51]. Indirect effects for PCV10 were examined in sensitivity analyses. Indirect effects for PCV10 in these analyses were assumed to be similar to estimating direct effects for PNE and AOM, in that indirect effects for PCV10 were derived by adjusting the PCV7 indirect effects by serotype coverage pre-introduction of PCV7 relative to PCV10 current serotype coverage. These data are presented in Table 2.

**Table 2:** Indirect effects, immunogenicity, and indirect effect adjustments for PCV7, PCV10, and PCV13

	**Bacteremia**	**Meningitis**	**Inpatient PNE**	**Outpatient PNE**	**Mild AOM**	**Moderate/Severe AOM**	**Source(s)/ Assumption**
Indirect effects (years)							
PCV7							
0–2	0.68	0.68	0.07	0.16	0.21	0.15	[40,42,44,65]
3–4	0.68	0.68	0.00	0.00	0.00	0.00	
5–17	0.39	0.39	0.00	0.00	0.00	0.00	
18–64	0.47	0.47	0.26	0.00	0.00	0.00	
64+	0.36	0.36	0.00	0.00	0.00	0.00	
PCV10 (for Sensitivity analysis)^a,b^							
0–2	0.41	0.41	0.04	0.10	0.13	0.09	
3–4	0.43	0.43	0.00	0.00	0.00	0.00	
5–17	0.26	0.26	0.00	0.00	0.00	0.00	
18–64	0.39	0.39	0.22	0.00	0.00	0.00	
64+	0.28	0.28	0.00	0.00	0.00	0.00	
PCV13^1^							
0–2	0.59	0.59	0.06	0.14	0.18	0.13	
3–4	0.62	0.62	0.00	0.00	0.00	0.00	
5–17	0.30	0.30	0.00	0.00	0.00	0.00	
18–64	0.55	0.55	0.30	0.00	0.00	0.00	
64+	0.42	0.42	0.00	0.00	0.00	0.00	
Immuno-genicity for PCV10 Direct Effects	0.90	0.90	0.85	0.80	0.75	0.75	[36]

*AOM* acute otitis media; *PCV7* 7-valent pneumococcal polysaccharide-protein conjugate vaccine; *PCV10* 10-valent pneumococcal conjugate vaccine; *PCV13* 13-valent pneumococcal conjugate vaccine; *PNE* pneumonia.

^a^ Adjusted for serotype coverage and year of coverage.

^b^ Indirect effects for PCV10 are assumed to be 0 in the base case.

### Economic inputs

#### Costs

The model considered direct and indirect medical costs necessary to treat each case of disease, each myringotomy, and any vaccine-acquisition costs. Direct medical costs were assumed to include diagnostics, physician time, hospitalization, prescriptions, and over-the counter medications as needed. These costs (Table 1) were obtained from Morrow et al. [19] and inflated to 2010 values using the Canadian-specific consumer price index multiplier [52].

Vaccination costs were based on the acquisition cost per dose plus a direct administration fee. List price acquisition cost per dose was assumed to be $86.26 for PCV13. Because acquisition cost of PCV10 was not available as of the writing of this article, the cost was assumed to be similar to the cost of PCV13. Administration cost per dose was estimated as $7.84 [52,53]. The impact of vaccine costs was examined in sensitivity analysis; a breakeven analysis is presented.

#### Utilities

Utility weights allow for an objective measurement of the desirability of a health state in a cost-utility analysis. A utility of 1.0 represents perfect health, whereas a value of 0.0 represents death. When combined with life-years, utilities produce quality-adjusted life-years (QALYs).

General health utility was obtained for the general Canadian population from the Canadian Community Health Survey [54]. This general health utility was calculated as weighted average of portion of individuals in each age group, and utility values reported. General health utilities for each age group are presented in Table 1.

Utility decrements due to neurological impairment and hearing loss were obtained from Morrow et al. [19] and were assumed to be 0.6 and 0.8, respectively. These decrements were similar to those used in previous Canadian specific analyses [13,14].

### Model calculations

The model presented the following annual outcomes: number of disease cases, sequelae, deaths, and costs. In addition, population life-years and QALYs were calculated along with incremental cost per QALY, which was calculated as the difference in total costs between PCV13 and PCV10 divided by the difference in QALYs in populations in which a PCV10 versus a PCV13 vaccination policy was implemented. If a strategy was less costly and more effective, it was a dominant strategy, referred to as cost-saving.

Several sensitivity and scenario analyses were performed. Specifically, we examined the impact of the following parameters on results: reduction in the percentage of children vaccinated, reduced immunogenicity of PCV10 direct effects, full indirect effects for PCV10, indirect effects for PCV10 at 50% of indirect effects that might occur, combined reduced immunogenicity of PCV10 direct effects and indirect effects at 50% of expected occurrence, combined reduced immunogenicity of PCV10 direct effects and indirect effects at 100% of expected occurrence, no direct effects on NTHi for PCV10, PCV13 indirect effects at 50% of indirect effects that might occur, increase in serotype coverage for PCV10 when accounting for cross-reactively with 6A, incidence of IPD and AOM as reported by Morrow et al. [19], serotype coverage as reported by Institut National de Sante Publique du Quebec [18], serotype coverage as reported for Alberta by Kellner et al. [2], direct effects for IPD as reported for a 2 + 1 dosing by Deceuninck et al. [34], 3 + 1 dosing schedule for PCV10, inclusion of indirect medical costs, and mortality as reported in Chuck et al. [14]. Results are presented in figure format.

## Results

### Base-case analysis

Table 3 presents the annual number of disease cases that would occur in Canada under each vaccination policy when considering only direct effects and direct and indirect effects.

**Table 3:** Annual number of disease cases in canada under each vaccine program

**Program**	**IPD**		**PNE**		**AOM**	
	**Bacteremia**	**Meningitis**	**In-patient**	**Out-patient**	**Mild**	**Moderate/Severe**
Direct and indirect effects						
PCV13	10,427	368	101,325	257,356	762,343	118,999
PCV10	14,511	512	110,085	263,557	789,344	122,150
Direct effects only						
PCV13	14,459	510	108,785	262,485	785,018	121,437
PCV10	14,511	512	110,085	263,557	789,344	122,150

*AOM* acute otitis media; *IPD* invasive pneumococcal disease; *PCV10* 10-valent pneumococcal conjugate vaccine; *PCV13* 13-valent pneumococcal conjugate vaccine; *PNE* pneumonia.

We observed 49,340 more cases of disease were prevented with PCV13 when both direct and indirect effects were considered. Specifically, 4,084 cases of bacteremia, 144 of meningitis, 8,760 of inpatient PNE, 6,201 of outpatient PNE, 27,001 of mild AOM, and 3,150 of moderate/severe AOM were prevented annually when vaccinating children with PCV13. This translated to 49 more myringotomies and 29 disease sequelae being prevented annually when both direct and indirect effects were considered. As a result, the population gained 15,283 more life-years and 13,828 more QALYs when vaccinating children with PCV13 versus PCV10. Approximately 879 deaths could be prevented annually and annual direct medical costs were reduced by $132.8 million when vaccinating children with PCV13 versus than PCV10. Thus, PCV13 was found to dominate PCV10. Results are presented in Table 4.

**Table 4:** Base-case cost and outcomes when considering both direct and indirect effects

**Outcome**	**PCV13**	**PCV10**	**Difference(PCV13 – PCV10)**
Total life-years	605,602,153	605,586,870	15,283
Total QALYs	543,278,132	543,264,303	13,828
Annual number of cases			
Myringotomy	1,868	1,918	−49.5
Neurological impairment	25.7	35.	−10.1
Hearing loss	47.8	66.5	−18.7
Death	9,601.03	10,480.47	−879.4
Annual direct medical costs	$1,185,354,128	$1,318,177,638	–$132,823,511
Cost per QALY			PCV13 dominates PCV10

*PCV10* 10-valent pneumococcal conjugate vaccine; *PCV13* 13-valent pneumococcal conjugate vaccine; *QALY* quality-adjusted life-year.

When considering direct effects only, we observed 7,466 more cases of disease (52 more cases of bacteremia, 2 of meningitis, 1,300 of inpatient PNE, 1,072 of outpatient PNE, 4,327 of mild AOM, and 713 of moderate/severe AOM) were prevented annually when vaccinating children with PCV13 versus PCV10. This translated to 11 more myringotomies and 0.3 more disease sequelae of neurological impairment and hearing loss prevented annually. As a result, the population gained 287 more life-years and 258 more QALYs when vaccinating children with PCV13 versus PCV10. Approximately 14 deaths could be prevented annually and annual direct medical costs (including the cost of vaccination) were reduced by $5.7 million when vaccinating children with PCV13 versus than PCV10. Thus, PCV13 was found to dominate PCV10. Results are presented in Table 5.

**Table 5:** Scenario analysis cost and outcomes when considering direct effects only

**Outcome**	**PCV13**	**PCV10**	**Difference (PCV13 – PCV10)**
Total life-years	605,587,157	605,586,870	287.45
Total QALYs	543,278,132	543,264,303	257.70
Annual number of cases			
Myringotomy	1,906	1,918	−11.0
Neurological impairment	35.7	35.8	−0.1
Hearing loss	66.3	66.5	−0.2
Death	10,466.50	10,480.47	−14.0
Annual direct medical costs	$1,312,418,621	$1,318,177,638	–$5,759,017
Cost per QALY			PCV13 dominates PCV10

*PCV10* 10-valent pneumococcal conjugate vaccine; *PCV13* 13-valent pneumococcal conjugate vaccine; *QALY* quality-adjusted life-year.

**Figure 2:**
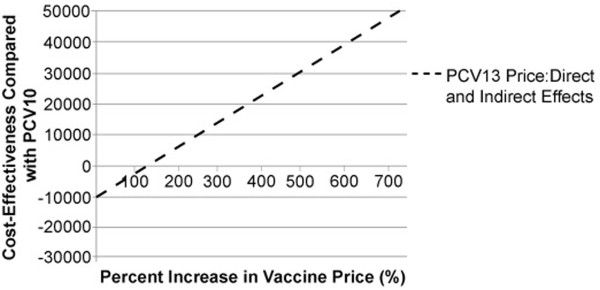
**Breakeven Analysis.** PCV10 = 10-valent pneumococcal conjugate vaccine; PCV13 = 13-valent pneumococcal conjugate vaccine. Dashed line represents the percentage increase in PCV13 cost per dose over PCV10 cost per dose that could occur at various cost-effectiveness thresholds when direct effects and indirect effects are considered.

Assuming a total per-dose cost of PCV10 of $94.10 (acquisition and administration) and considering a threshold incremental cost per QALY of $50,000, the cost per dose for PCV13 could be as high as $737.11 to remain cost-effective, when both direct and indirect effects were considered. PCV13 is cost neutral at a cost per dose of $197.72 or at a 110% increase in cost per dose. The percentages of allowable price increases in PCV13 vaccine over PCV10 vaccine in order to maintain various cost-effectiveness levels is presented in Figure 2. We also examined the effect of reducing the cost per dose of PCV10 while maintaining a cost per dose of $94.10 for PCV13 and found that even if the cost per dose of PCV10 were $0, cost neutrality still could not be achieved (data no shown).

### Sensitivity analyses

**Figure 3:**
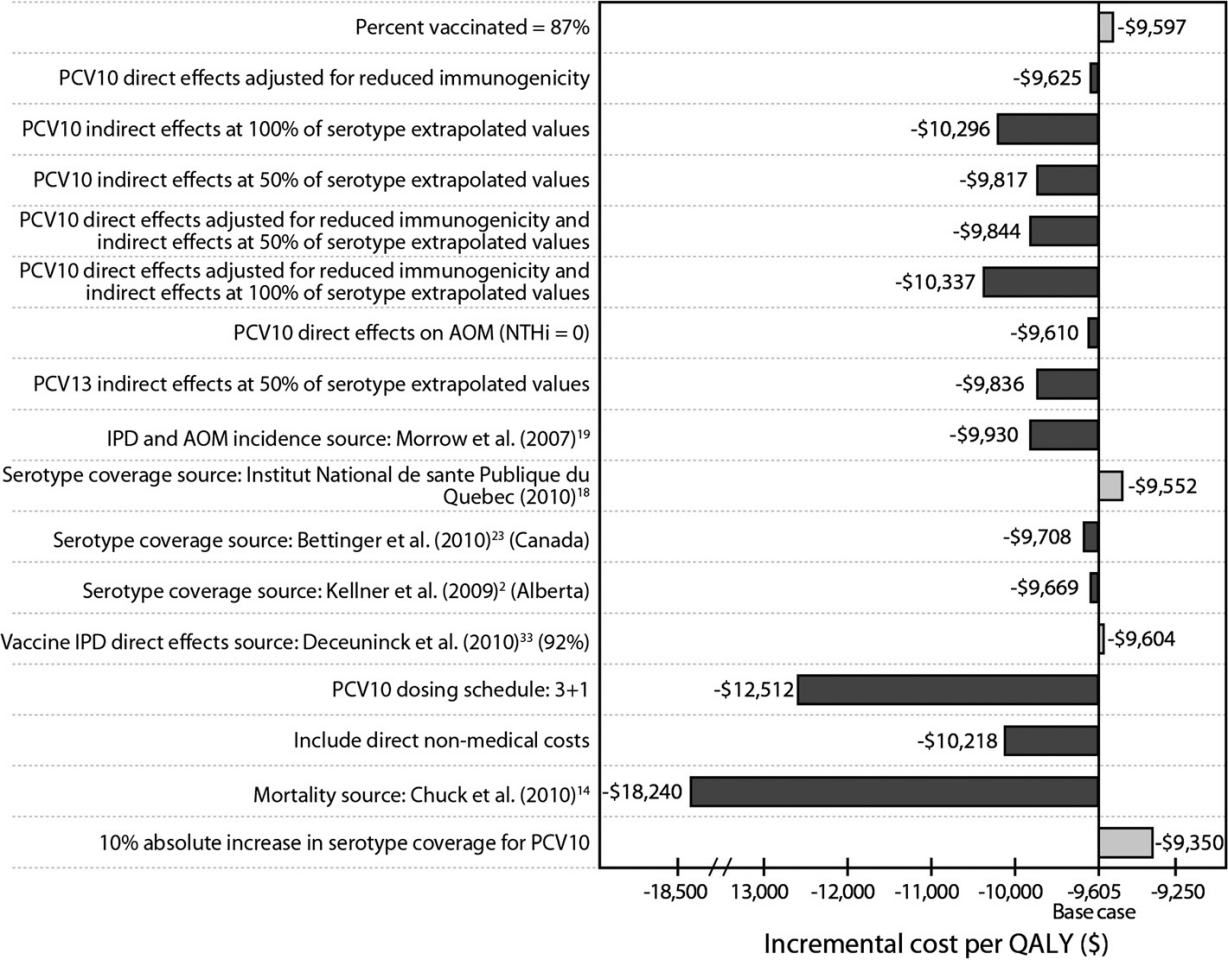
**Sensitivity analysis results (Based on a base case of direct and indirect effects being assumed).** AOM = acute otitis media; IPD = invasive pneumococcal disease; NTHi = non-typeable *Haemophilus influenzae*; PCV10 = 10-valent pneumococcal conjugate vaccine; PCV13 = 13-valent pneumococcal conjugate vaccine; QALY = quality-adjusted life-year. Dark shaded bar represents an increase in value for the denoted parameter. Light shaded bar represents a decreased in value for the denoted parameter.

Overall, in all scenarios of sensitivity analysis, PCV13 prevented more cases of disease and remained cost-saving compared with PCV10 (Figure 3). Using serotype coverage as reported by Bettinger et al. [24] showed PCV13 to gain the highest total QALYs (17,956), whereas assuming indirect effect for PCV10 similar to that seen for PCV7 showed the lowest total gain in QALYs (5,102). In these scenarios, the greatest ($174,312,074) and the lowest ($62,824,747) reduction in total annual costs were observed, respectively. Changes in overall QALYs gained and annual costs saved for all other scenario fell between these ranges.

## Discussion

We developed a decision-analytic model to compare the costs and outcomes associated with a national immunization program of PCV13, as is currently implemented throughout Canada, compared with PCV10, which was previously used in some provinces following usage of PCV7 and before transitioning to PCV13. Overall, widespread use of PCV13 in Canada was expected to have a substantial public-health impact and be cost-saving due to its demonstrated immunogenicity and broader serotype coverage when compared with PCV10.

Our analysis approach was similar to other Canadian-specific analyses in the literature, in terms of the decision-analytic model structure, diseases considered, and approaches to input data [13,14,25,31]. Talbird et al. performed an all-Canada analysis that compared the use of PCV10 to PCV7. Thus, it was difficult compare results of our analysis to that analysis. Although we saw many similarities in the input data (i.e., incidence, mortality, costs, sequelae utilities), Talbird and colleagues drew their serotype coverage data from a different surveillance-coordinating center and estimated serotype coverage as an average over a 10-year period. Conversely, we considered current serotype coverage as reported by the National Centre for Streptococcus. In addition, Talbird et al. considered PCV10 direct effects on NTHi in IPD and AOM, in which the effect of PCV10 on NTHi-caused AOM was substantial (30%–38% of AOM due to *Streptococcus pneumonia* and 38%–57% of non-*Streptococcus pneumonia* disease due to NTHi) [31]. Talbird et al. also considered a utility decrement due to having AOM, making the approach in our analysis more conservative [55].

Chuck et al. [14] compared the use of PCV10 to PCV13 in Alberta. Their results differed slightly in that they found PCV10 to be cost-effective when considering coverage of NTHi in AOM. Although our analyses were similar around assumptions on utilities, incidence of disease sequelae, and costs, Chuck et al. assumed a greater rate of mortality. In addition, Chuck and colleagues considered an incidence combined with serotype coverage in Alberta as estimated from the National Centre for Streptococcus. An in-depth comparison of the incidence and serotype coverage data values found that the incidence along with serotype coverage assumed in their analysis was much lower than assumed in our analysis. A comparison of these data values with Talbird et al. showed our data values to be more in line with Talbird et al. [31]. It is unclear whether the incidence along with serotype coverage was much lower in Alberta than for the rest of Canada. As a result, the value of PCV13 could be overestimated. All in all, to account for these differences, we performed multiple scenario analyses around our assumptions on both incidence and serotype coverage. It will be important for decision makers to examine these analyses closely and determine which analysis most closely resembles their situation.

**Figure 4:**
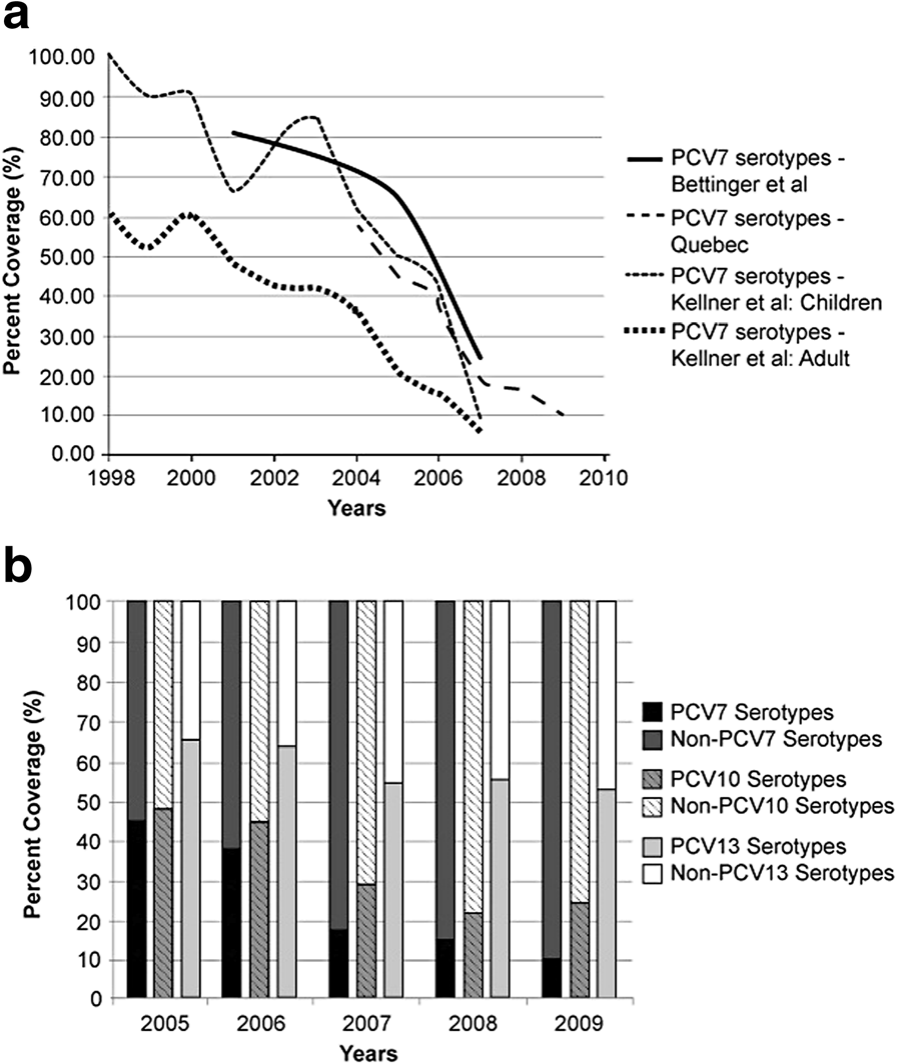
**Change in PCV7 and non-PCV7 serotype coverage over time. (a)** Change in PCV7 Serotype Coverage Over Time. PCV7 = 7-valent pneumococcal polysaccharide-protein conjugate vaccine. Solid line represents coverage of PCV7 serotypes for Canada as reported by Bettinger et al. (2010). Dashed line represents coverage of PCV7 serotypes for Quebec the Institut National de Sante Publique du Quebec (2010). Dashed-dotted line represents coverage of PCV7 serotypes for children in Alberta as reported by Kellner et al. (2009). Square-dotted line represents coverage of PCV7 serotypes for adults in Alberta as reported by Kellner et al. (2009). **(b)** Serotype Coverage for All Pneumococcal Vaccines Over Time. PCV7 = 7-valent pneumococcal polysaccharide-protein conjugate vaccine; PCV10 = 10-valent pneumococcal conjugate vaccine; PCV13 = 13-valent pneumococcal conjugate vaccine. Dark solid bar represents PCV7 serotype. Medium solid bar represents non-PCV7 serotypes. Dark striped bar represents PCV10 serotypes. Light striped bar represents non-PCV10 serotypes. White-shaded bar represents PCV13 serotypes. Light-shaded bar represents non-PCV13 serotypes.

The disease epidemiology has changed dramatically since the introduction of PCV7 in 2003 [1-8]. Specifically, there has been a fair amount of serotype replacement. Figure 4 shows the trends of PCV7-covered serotypes over time, as measured by a number of surveillance studies. As seen in Figure 4a, while PCV7-covered serotypes have decreased approximately 6-fold, PCV7 non-covered serotypes have increased 2- to 4-fold in various Canadian territories and provinces. From the Institut National de Sante Publique du Quebec, we also observed that in the early years of PCV7, PCV7 covered approximately 50% of pneumococcal disease (Figure 4b) [18]. Over the years and because of the implementation of PCV7, PCV7-covered serotypes have been dramatically reduced. Examining current serotype coverage, we observed that PCV13 was estimated to cover approximately 50% of pneumococcal disease. In addition, data from Laboratoire De La Sante Publique de Quebec for 2009 showed 19A as the most prevalent serotype across all ages, covering nearly 18% of all serotype isolates in Quebec and representing almost 68% of vaccine-preventable IPD covered by PCV 13. Data from the National Centre for Streptococcus for 2009–2010 confirmed 19A to be the most prevalent among 0- to 4-year-olds [23]. With PCV13 being the only vaccine to cover 19A, significant reductions in disease might be expected. Sensitivity analysis using the various serotype coverage estimates showed PCV13 to remain cost-saving when compared with PCV10.

PCV10 has been shown to have a lower immunogenic response than PCV7 among the seven common serotypes, as demonstrated by lower geometric mean antibody concentrations and by percentage of infants achieving serum immunoglobulin G seropositivity ≥ 0.20 μg/mL [10]. In our base-case analysis, we did not consider the lower immunogenic effect of PCV10. However, in sensitivity analysis we examined the impact of reduced effectiveness and showed that PCV13 continued to dominate PCV10. PCV7 consistently demonstrated a statistically significant reduction in nasopharyngeal carriage [45,56,57], which is a critical component to creating an indirect effect. The data on statistically significant reduction in nasopharyngeal carriage is now emerging [48]. PCV10 has not demonstrated this reduction [10,51]. As a result, we assumed no indirect effects to PCV10 in the base case. It is possible that PCV10 could exhibit indirect effects. Thus, we examined the presence and absence of indirect effects for PCV10 and PCV13, respectively, in sensitivity analyses. In all analyses, PCV13 was found to remain cost-saving compared with PCV10.

In cost-effectiveness analyses of pneumococcal conjugate vaccines, assumptions around AOM incidence and efficacy played an important role in the direct medical costs accrued. Although the costs associated with a single AOM event were low relative to hospitalized disease, due to the high incidence of AOM these costs were a substantial contributor to the disease burden calculated within our model. In addition, because the etiology of AOM is typically all-cause, a critical component to the cost-effectiveness of pneumococcal vaccines was the impact of vaccine effectiveness on NTHi. Recent economic analyses have shown that PCV10 reduces costs and is cost-effective when effectiveness on NTHi is considered [14,58]. Chuck et al. [14] assumed that PCV10 would have a 3% increase in direct effects on all AOM due to NTHi. However, Talbird et al. presented an etiology-weighted estimate of 22.9% reduction in all-cause AOM relative to 6.7% for PCV7. Subsequent analyses also used this approach [58,59]. Details of this approach have been discussed elsewhere [55,60].

In this analysis, we considered both direct effects and a combination of direct and indirect effects of the vaccines. However, evidence of indirect effects for PCV10 and PCV13 are limited in the literature. Indirect effects for PCV7 have been firmly established in the Canada, the US, Australia, and the UK [2,24,45,46,61-63]. Because PCV13 shares the same carrier protein as PCV7, we assumed PCV13 would incur similar indirect effects as PCV7. In addition, a recent analysis performed by the CDC demonstrated a decline in PCV13-type IPD in adults in the US [49]. However, we acknowledge that a recent presentation by De Wals and colleagues [64] noted that the existence of indirect effects in the presence of PCV7 for all-cause PNE could not be identified in Quebec. An indirect effect for IPD in adults was observed, but the authors also observed “strong and rapid” serotype replacement. Thus, a targeted vaccination program in adults may provide better protection to this group [64]. We examined of the use of PCV10 and PCV13 in the presence and absence of indirect effects and found PCV13 to be cost-saving in both scenarios. We acknowledge that we did not explicitly examine the impact of serotype replacement following the use of higher-valent conjugate vaccines. As these data become available, it will be important to consider this impact.

A limitation of this model is the uncertainty around vaccine prices due to the confidential nature of tender negotiations. When including indirect effects, our model was not sensitive to PCV13 price variation; PCV13 remained a cost-saving option even if PCV13 was twice the price of PCV10. In addition, PCV13 remained cost-saving regardless of the reduction in cost per dose for PCV10 when the cost per dose of PCV13 was held constant. This was due to the substantial burden of disease caused by PCV13-specific serotypes compared with PCV10-covered serotypes. This difference in coverage represents more than 27% of all serotypes in Quebec [18]. Due to the confidential nature of negotiated tenders, it will be important for decision-makers to perform these analyses under their own pricing scenarios so that they can better understand the economic value of PCV13 compared with PCV10.

## Conclusions

In conclusion, our analysis shows PCV13 to be cost-saving compared with PCV10, given the most recently available epidemiology of disease and the potential effects of PCV10 and PCV13. Because of the burden of disease that is caused by serotype 19A, provinces and territories have adopted the use of PCV13. Further research will be needed to monitor the effect of PCV13 on disease in Canada, including the effect on 19A in vaccinated and unvaccinated populations and on emergent non-vaccine serotypes. However, health directors in provinces and territories should be assured that they have implemented a cost-savings policy.

## Abbreviation

AOM = acute otitis media; IPD = invasive pneumococcal disease; NTHi = non-typeable Haemophilus influenza; PCV7 = 7-valent pneumococcal polysaccharide-protein conjugate vaccine; PCV10 = 10-valent pneumococcal conjugate vaccine; PCV13 = 13-valent pneumococcal conjugate vaccine; PNE = pneumonia; QALY = quality-adjusted life-year.

## Competing interests

This study was sponsored by Pfizer Canada, Inc. Giovanni Zanotti is an employee of Pfizer Canada, Inc., and Raymond A. Farkouh is an employee of Pfizer Inc., which manufacturers 7-valent and 13-valent pneumococcal polysaccharide-protein conjugate vaccines. Stephanie R. Earnshaw and Cheryl L. McDade are employees of RTI Health Solutions, an independent contract research organization that has received research funding from Pfizer Canada, Inc. for this and other research studies, as well as funding from other pharmaceutical companies that market vaccines and drugs for other medical conditions.

## Authors’ contributions

SRE conceptualised the design of the study, acquired data, performed analysis, and interpreted data. She also drafted the manuscript, reviewed and revised the manuscript critically for important content, and approved the final manuscript. CM was involved in the conceptualisation of the design of the study, acquired data, programmed the decision model, performed the analysis, reviewed and revised the manuscript critically for important content, and approved the final manuscript. GZ was involved in acquisition of data, interpretation of data, review and revision of the manuscript critically for important content, and approved the final manuscript. RAF was involved in interpretation of data, review and revision of the manuscript critically for important content, and approved the final manuscript. DS was involved in the conceptualisation of the design of the study, interpretation of data, and approved the final manuscript.

## Pre-publication history

The pre-publication history for this paper can be accessed here:

http://www.biomedcentral.com/1471-2334/12/101/prepub

## Supplementary Material

Additional file 1 Table A-1. Sensitivity Analysis Input Parameters. Click here for file
