# Supplementary material for: Cost-effectiveness of 2 + 1 dosing of 13-valent and 10-valent pneumococcal conjugate vaccines in Canada
Source: BMC Infect Dis. 2012 Apr 24;12:101. doi: 10.1186/1471-2334-12-101 (PMC3532329; doi:10.1186/1471-2334-12-101)
Supplement: Additional file 1 — Table A-1. Sensitivity Analysis Input Parameters. [file 1471-2334-12-101-S1.docx]

Tables for Online Appendix

Table A-1. Sensitivity Analysis Input Parameters

| Parameters | Age (Years) | | | | | Source |
| --- | --- | --- | --- | --- | --- | --- |
|  | 0-2 | 2-4 | 5-17 | 18-64 | 65+ |  |
| Incidence (per 100,000) |  |  |  |  |  |  |
| Bacteremia | 481.33 | 332.67 | 235.56 | 1297.44 | 1428.00 | Morrow et al. (2007); Ray et al. (2006) |
| Meningitis | 72.00 | 16.00 | 22.22 | 70.78 | 45.00 |  |
| Inpatient PNE | 1406.33 | 1174.67 | 1024.67 | 2752.33 | 6469.00 |  |
| Mild AOM | 184951.97 | 159507.68 | 136750.96 | 0.00 | 0.00 |  |
| Moderate/severe AOM | 20142.03 | 9394.32 | 8054.04 | 0.00 | 0.00 |  |
| Serotype Coverage |  |  |  |  |  |  |
| Quebec |  |  |  |  |  |  |
| PCV10 | 26.39% | 26.39% | 26.39% | 26.39% | 26.39% | Institut National de Sante Publique du Quebec (2010) |
| PCV13 | 53.88% | 53.88% | 53.88% | 53.88% | 53.88% |  |
| Alberta |  |  |  |  |  | Kellner et al. (2009) |
| PCV10 | 33.33% | 33.33% | 57.71% | 57.71% | 57.71% |  |
| PCV13 | 66.67% | 66.67% | 66.86% | 66.86% | 66.86% |  |
| Canada |  |  |  |  |  | Bettinger et al. (2010) |
| PCV10 | 38.03% | 38.03% | 38.03% | 38.03% | 38.03% |  |
| PCV13 | 71.86% | 71.86% | 71.86% | 71.86% | 71.86% |  |
| Mortality |  |  |  |  |  |  |
| Inpatient PNE | 0.01 | 0.01 | 0.011385 | 0.016 | 0.016 | Chuck et al. (2010) |
| Direct Non-medical Costs |  |  |  |  |  |  |
| Bacteremia | $544.78 | $272.39 | $726.37 | $499.38 | $408.58 | Morrow et al. (2007); Statistics Canada (2011) |
| Meningitis | $1,265.86 | $632.93 | $1,687.81 | $1,160.37 | $949.39 |  |
| Inpatient PNE | $422.48 | $211.24 | $563.31 | $387.27 | $316.86 |  |
| Outpatient PNE | $122.30 | $61.15 | $163.06 | $112.11 | $91.72 |  |
| Mild AOM | $71.47 | $35.74 | $53.60 | $0.00 | $0.00 |  |
| Moderate/severe AOM | $71.47 | $35.74 | $53.60 | $0.00 | $0.00 |  |

AOM = acute otitis media; PCV10 = 10-valent pneumococcal conjugate vaccine; PCV13 = 13-valent pneumococcal conjugate vaccine; PNE = pneumonia.

Caption: Sensitivity analysis input parameters used for PCV13 analysis.
